# Supplementary material for: Enhancing climate resilience with proximal cues in personalized climate disaster preparedness messaging
Source: Nat Hum Behav. 2025 Dec 8;10(3):505–13. doi: 10.1038/s41562-025-02352-w (PMC13017516; doi:10.1038/s41562-025-02352-w)
Supplement: Supplementary file 1 — Appendices A–E. [file 41562_2025_2352_MOESM1_ESM.pdf]

nature human behaviour

Supplementary information https://doi.org/10.1038/s41562-025-02352-w

Enhancing climate resilience with proximal cues in personalized climate disaster preparedness messaging

In the format provided by the authors and unedited

Table of Contents

Appendix A: Intervention Texts 2

Appendix B: Full Email Send-out Image 3

Appendix C: Bushfire Preparedness Landing Page 4

Appendix D: Linear Regression Results 5

Appendix E: Exploratory Moderation Regression Results.. 6

Appendix A: Intervention Texts

	Control	Treatment
**Subject line**	<First name>, prepare ahead to help keep your property safe	<First name>, prepare ahead to keep your property in <suburb> safe
**Pre-header**	It’s important to plan ahead	
**Header**	Prepare your property in case of bushfires.	Prepare your property in <suburb> in case of bushfires.
**Intro**	Hi <first name>,<br>It’s important to take steps to ensure your property is prepared for extreme weather, especially during the warmer months. According to the NSW Rural Fire Service, your property in <suburb> may be located in a bushfire prone area. It’s best to stay on top of routine maintenance to take care of your property and to help keep you safe.	
**Tips**	• Clear and clean your gutters: embers and sparks can quickly set leaves alight – so it’s a good idea to clear your roof, gutters, and downpipes of debris.<br>• Maintain your garden and fencing: cutting back trees or shrubs on your property and keeping your lawn well-maintained can help minimise the spread of fire.<br>• Remove flammable items: by removing these items you are reducing potential fuel for bushfires. These can include rubbish, gas bottles and paint cans.<br><br>For more tips to help you prepare your property, click on the button below or visit bankname.com.au/prepareahead	

Appendix B: Full Email Send-out Image

Control	Treatment

Control

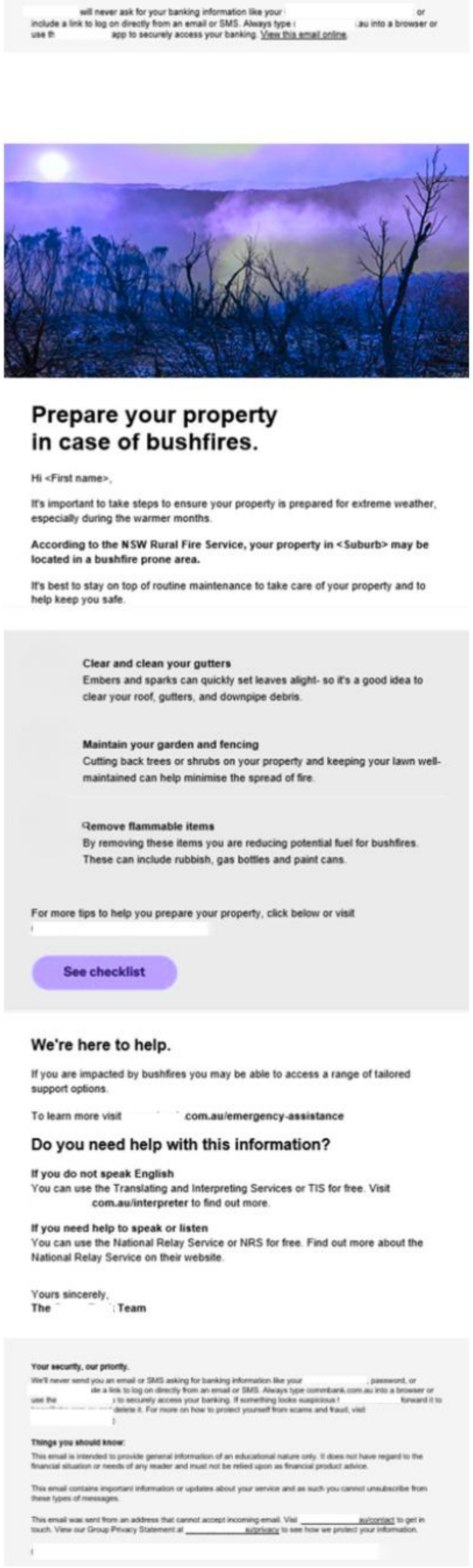

will never ask for your banking information like your or include a link to log on directly from an email or SMS. Always type .au into a browser or use th app to securely access your banking. View this email online

Prepare your property in case of bushfires.

Hi <First name>,

It's important to take steps to ensure your property is prepared for extreme weather, especially during the warmer months.

According to the NSW Rural Fire Service, your property in <Suburb> may be located in a bushfire prone area.

It's best to stay on top of routine maintenance to take care of your property and to help keep you safe.

Clear and clean your gutters
Embers and sparks can quickly set leaves alight- so it's a good idea to clear your roof, gutters, and downpipe debris.

Maintain your garden and fencing
Cutting back trees or shrubs on your property and keeping your lawn well-maintained can help minimise the spread of fire.

Remove flammable items
By removing these items you are reducing potential fuel for bushfires. These can include rubbish, gas bottles and paint cans.

For more tips to help you prepare your property, click below or visit

See checklist

We're here to help.

If you are impacted by bushfires you may be able to access a range of tailored support options.

To learn more visit **.com.au/emergency-assistance**

Do you need help with this information?

If you do not speak English
You can use the Translating and Interpreting Services or TIS for free. Visit **com.au/interpreter** to find out more.

If you need help to speak or listen
You can use the National Relay Service or NRS for free. Find out more about the National Relay Service on their website.

Yours sincerely,
The Team

Your security, our priority.
We'll never send you an email or SMS asking for banking information like your , password, or de a link to log on directly from an email or SMS. Always type commbank.com.au into a browser or use the to securely access your banking. If something looks suspicious forward it to delete it. For more on how to protect yourself from scams and fraud, visit

Things you should know:
This email is intended to provide general information of an educational nature only. It does not have regard to the financial situation or needs of any reader and must not be relied upon as financial product advice.

This email contains important information or updates about your service and as such you cannot unsubscribe from these types of messages.

This email was sent from an address that cannot accept incoming email. Visit au/contact to get in touch. View our Group Privacy Statement at au/privacy to see how we protect your information.

Treatment

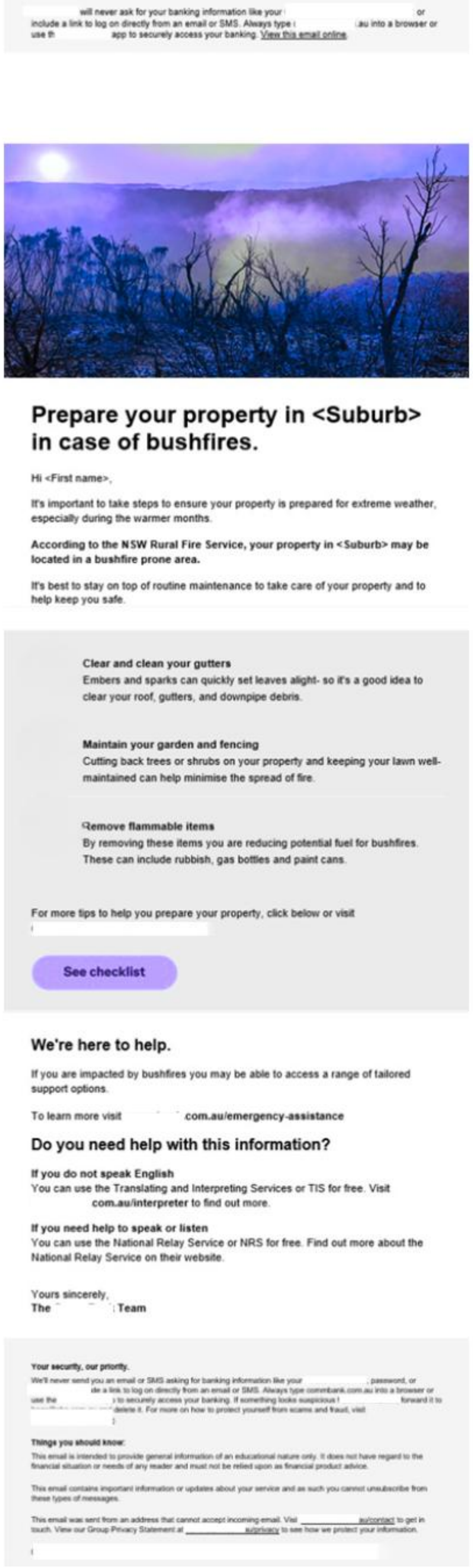

will never ask for your banking information like your or include a link to log on directly from an email or SMS. Always type .au into a browser or use th app to securely access your banking. View this email online

Prepare your property in <Suburb> in case of bushfires.

Hi <First name>,

It's important to take steps to ensure your property is prepared for extreme weather, especially during the warmer months.

According to the NSW Rural Fire Service, your property in <Suburb> may be located in a bushfire prone area.

It's best to stay on top of routine maintenance to take care of your property and to help keep you safe.

Clear and clean your gutters
Embers and sparks can quickly set leaves alight- so it's a good idea to clear your roof, gutters, and downpipe debris.

Maintain your garden and fencing
Cutting back trees or shrubs on your property and keeping your lawn well-maintained can help minimise the spread of fire.

Remove flammable items
By removing these items you are reducing potential fuel for bushfires. These can include rubbish, gas bottles and paint cans.

For more tips to help you prepare your property, click below or visit

See checklist

We're here to help.

If you are impacted by bushfires you may be able to access a range of tailored support options.

To learn more visit **.com.au/emergency-assistance**

Do you need help with this information?

If you do not speak English
You can use the Translating and Interpreting Services or TIS for free. Visit **com.au/interpreter** to find out more.

If you need help to speak or listen
You can use the National Relay Service or NRS for free. Find out more about the National Relay Service on their website.

Yours sincerely,
The Team

Your security, our priority.
We'll never send you an email or SMS asking for banking information like your , password, or de a link to log on directly from an email or SMS. Always type commbank.com.au into a browser or use the to securely access your banking. If something looks suspicious forward it to delete it. For more on how to protect yourself from scams and fraud, visit

Things you should know:
This email is intended to provide general information of an educational nature only. It does not have regard to the financial situation or needs of any reader and must not be relied upon as financial product advice.

This email contains important information or updates about your service and as such you cannot unsubscribe from these types of messages.

This email was sent from an address that cannot accept incoming email. Visit au/contact to get in touch. View our Group Privacy Statement at au/privacy to see how we protect your information.

Appendix C: Bushfire Preparedness Landing Page

How to prepare your home and car for a bushfire

Bushfires can be unpredictable and fast-moving, often leaving very little time to make vital decisions. It's ir before each bushfire season and prepare your home and car to help keep you and your family safe.

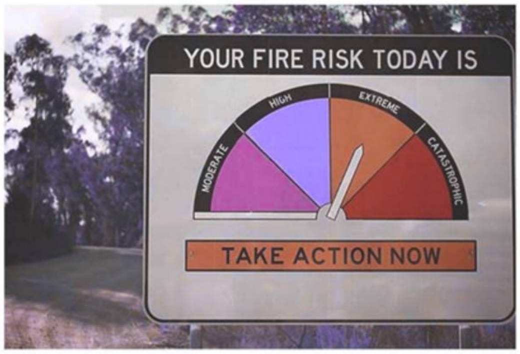

If you live in a bushfire prone area, there are simple ways you can help protect your home and family before the start of the season.

Plan ahead by documenting a bushfire survival plan, clearing your garden of flammable materials and leaf litter, and reviewing your home and car insurance documents to ensure you have the right level of cover.

Safety guidance and emergency contact information may differ state by state. Please refer to the Bureau of Meteorology website to learn more about how to prepare for a fire where you live.

1. Create a bushfire plan

You may want to create a bushfire survival plan and discuss it with every member of the household, so each person knows exactly what to do in case of a fire.

A bushfire plan includes knowing the risk in the area you live, preparing the home and creating an equipment and action checklist that anyone in the home can follow.

For more information about bushfire preparation and creating a plan, check out the NSW Rural Fire Service's Bushfire Survival Plan.

Remember, it's also important to regularly check, test and update your fire safety equipment in the home, like your smoke alarms and fire blankets.

2. Clear out your gutters

Embers and sparks can quickly set leaves alight – so it's a good idea to engage a professional gutter cleaning service to clear your roof, gutters and downpipes of debris.

For extra fireproofing, you could also attach a sprinkler system to the gutters and install gutter guards which can help prevent leaves from gathering.

3. Maintain your garden and fencing

Cutting back any trees or shrubs near buildings and keeping your lawn well-maintained can help minimise the spread of fire. Check with your local council on the regulation on trees as it varies by state. Don't forget to clean up fallen leaves, twigs and debris around the property.

If you're in a bushfire prone area, consider:

- Breaking up your garden with decorative paving
- Where you can, opt for metal fences which can better withstand extreme heat
- Ensuring an outdoor water supply is accessible, like a water tank or sprinkler system
- Checking your garden hose is long enough to reach the entire perimeter of your property

For more tips on preparing your property for a bushfire, you may like to consult the Country Fire Service (CFS) website.

4. Remove flammable items

Remove flammable items and hazards in your backyard to prevent providing fuel to bushfires. These can include mats and furniture on your veranda or deck, wood or rubbish piles, aerosols, paint cans and flammable liquids like petrol.

It's also important to keep an eye out for loose or damaged roof tiles and promptly repair gaps in your external walls to prevent embers from getting in.

5. Prepare your car

In a bushfire, your car can be an essential escape option. But remember, driving into a fire front is extremely dangerous, so plan to leave early.

If your car is garaged, make sure a garage door opener is part of your emergency kit, and that you are familiar with the manual opening mechanism if there is a power outage.

Be vigilant when travelling on the road during bushfire season. The Country Fire Service (CFS) website has tips on what to do if you're trapped by fire whilst in a vehicle.

6. Check your insurance cover

Regularly review your home and car insurance to ensure your cover is up to date and offers the right level of protection for your circumstances.

Understanding the inclusions and exclusions of fire cover in your policy can help in case you need to make a claim. For instance, does your policy cover loss or damage to building and contents caused by fire (including bushfire), or heat, smoke or soot as a result of fire?

Generally, there's a 48-hour waiting period after starting a policy before you can claim on bushfire damage. Check your policy, as waiting periods vary with each insurer.

Appendix D: Linear Regression Results

	B	SE	t(12983)	p	95% CI
**Landing page clicks**					
Condition	0.005	0.001	3.274	.001	0.002, 0.008
Intercept	0.004	0.001	4.553	< .001	0.003, 0.007
**Landing page visits**					
Condition	0.002	0.001	2.771	.005	0.001, 0.004
Intercept	0.001	0.001	1.385	.166	-0.0003, 0.002
**Email openings**					
Condition	0.017	0.008	1.963	.049	0.0001, 0.035
Intercept	0.534	0.006	85.977	< .001	0.522, 0.546

Appendix E: Exploratory Moderation Regression Results

Moderator	Condition Effect B (95% CI)	Moderator Effect B (95% CI)	Interaction Effect B (95% CI)	Interaction *p*-value	Interaction Sig.
Gender	0.964 [0.233, 1.695] (p = .0097)	0.715 [-0.040, 1.470] (p = .063)	-0.404 [-1.313, 0.505]	.383	No
Age	0.008 [-1.778, 1.794] (p = .993)	0.023 [-0.005, 0.051] (p = .098)	0.014 [-0.020, 0.048]	.428	No
Annual income	0.748 [-0.597, 2.093] (*p* = .276)	–2.85e–07 [-0.000, 0.000] (*p* = .952)	–2.99e–06 [-0.000, 0.000]	.702	No
Bank tenure	0.882 [-0.112, 1.876] (*p* = .082)	0.022 [-0.009, 0.052] (*p* = .166)	–0.008 [-0.045, 0.029]	.674	No
Checking balance	0.692 [0.240, 1.143] (p = .0027)	–1.20e–07 [-0.000, 0.000] (*p* = .873)	–1.99e–07 [-0.000, 0.000]	.823	No
Savings balance	0.704 [0.268, 1.139] (p = .0015)	–4.43e–07 [-0.000, 0.000] (*p* = .580)	–1.76e–07 [-0.000, 0.000]	.858	No
Credit card balance	0.699 [0.231, 1.166] (p = .0034)	6.40e–06 [-0.000, 0.000] (*p* = .830)	3.86e–06 [-0.000, 0.000]	.916	No
Home loan balance	0.414 [-0.207, 1.036] (*p* = .191)	–7.93e–07 [-0.000, 0.000] (*p* = .097)	6.41e–07 [-0.000, 0.000]	.225	No
Personal loan balance	0.708 [0.274, 1.142] (p = .0014)	–5.93e–05 [-0.000, 0.000] (*p* = .586)	–8.14e–06 [-0.000, 0.000]	.954	No

Note: Models are negative binomial regressions testing the effect of condition (treatment vs. control) on main outcome variables of clicks. No significant interactions were found, indicating the effect of the message did not significantly vary by demographic or financial characteristics.
