# Supplementary material for: Enhancing climate resilience with proximal cues in personalized climate disaster preparedness messaging
Source: Nat Hum Behav. 2025 Dec 8;10(3):505–13. doi: 10.1038/s41562-025-02352-w (PMC13017516; doi:10.1038/s41562-025-02352-w)
Supplement: Supplementary file 2 — Reporting Summary [file 41562_2025_2352_MOESM2_ESM.pdf]

nature portfolio

Corresponding author(s): Nurit Nobel

Last updated by author(s): Sep 9, 2025

Reporting Summary

Nature Portfolio wishes to improve the reproducibility of the work that we publish. This form provides structure for consistency and transparency in reporting. For further information on Nature Portfolio policies, see our Editorial Policies and the Editorial Policy Checklist.

Statistics

For all statistical analyses, confirm that the following items are present in the figure legend, table legend, main text, or Methods section.

n/a	Confirmed	
☐	☒	The exact sample size (n) for each experimental group/condition, given as a discrete number and unit of measurement
☐	☒	A statement on whether measurements were taken from distinct samples or whether the same sample was measured repeatedly
☐	☒	The statistical test(s) used AND whether they are one- or two-sided<br>*Only common tests should be described solely by name; describe more complex techniques in the Methods section.*
☒	☐	A description of all covariates tested
☒	☐	A description of any assumptions or corrections, such as tests of normality and adjustment for multiple comparisons
☐	☒	A full description of the statistical parameters including central tendency (e.g. means) or other basic estimates (e.g. regression coefficient) AND variation (e.g. standard deviation) or associated estimates of uncertainty (e.g. confidence intervals)
☐	☒	For null hypothesis testing, the test statistic (e.g. F, t, r) with confidence intervals, effect sizes, degrees of freedom and P value noted<br>*Give P values as exact values whenever suitable.*
☒	☐	For Bayesian analysis, information on the choice of priors and Markov chain Monte Carlo settings
☒	☐	For hierarchical and complex designs, identification of the appropriate level for tests and full reporting of outcomes
☐	☒	Estimates of effect sizes (e.g. Cohen's d, Pearson's r), indicating how they were calculated

Our web collection on statistics for biologists contains articles on many of the points above.

Software and code

Policy information about availability of computer code

Data collection	Experimental data was received from a large Australian Bank under a Research Partnership Agreement with Harvard STAR Lab.
Data analysis	Data analysis was conducted in the statistical software R (v.4.3.1). Code is available publicly at Research Box: https://researchbox.org/2804

For manuscripts utilizing custom algorithms or software that are central to the research but not yet described in published literature, software must be made available to editors and reviewers. We strongly encourage code deposition in a community repository (e.g. GitHub). See the Nature Portfolio guidelines for submitting code & software for further information.

Data

Policy information about availability of data

All manuscripts must include a data availability statement. This statement should provide the following information, where applicable:

- Accession codes, unique identifiers, or web links for publicly available datasets
- A description of any restrictions on data availability
- For clinical datasets or third party data, please ensure that the statement adheres to our policy

The data analyzed in this article were provided by the research partner (a large retail bank) and contain sensitive financial information. To protect participant privacy, and under a nondisclosure agreement, we are not authorized to publicly share the data. Under the terms of the bank's data-use agreement and applicable privacy laws, the underlying microdata cannot be shared with external parties. Interested researchers may contact the corresponding author at nurit.nobel@hhs.se

to obtain access to a replication package, with responses provided within 3 months. The package will include a synthetic dataset with coarsened data that mimics the structure and key summary statistics of the original data while containing no real customer records.

Research involving human participants, their data, or biological material

Policy information about studies with human participants or human data. See also policy information about sex, gender (identity/presentation), and sexual orientation and race, ethnicity and racism.

Reporting on sex and gender	Descriptive statistics are reported for gender as reported by participants upon enrolling as bank customers.
Reporting on race, ethnicity, or other socially relevant groupings	Race or ethnicity data was not collected in this experiment and hence not reported in the manuscript.
Population characteristics	See "Setting and Participants" and Table 1 for detailed participant characteristics.
Recruitment	See "Setting and Participants" for inclusion criteria in the sample.
Ethics oversight	The Harvard University Committee on the Use of Human Subjects (CUHS) approved the protocol of this study (reference #: IRB23-1716)

Note that full information on the approval of the study protocol must also be provided in the manuscript.

Field-specific reporting

Please select the one below that is the best fit for your research. If you are not sure, read the appropriate sections before making your selection.

☐ Life sciences ☒ Behavioural & social sciences ☐ Ecological, evolutionary & environmental sciences

For a reference copy of the document with all sections, see nature.com/documents/nr-reporting-summary-flat.pdf

Behavioural & social sciences study design

All studies must disclose on these points even when the disclosure is negative.

Study description	This is a field experiment, also known as Randomized Controlled Trial. It is a quantitative study.
Research sample	Participants were bank customers of our field partner, who have an active bank account (tenure with the bank between 1-64 years, M = 21.4, SD = 11.8), and who are homeowners whose property is located in an area determined to be prone to bushfires (wildfires) in New South Wales (NSW), Australia. Participants were between 19-96 years of age (M = 47.1, SD = 12.5), and 49.4% were female.
Sampling strategy	Bushfire risk was determined by the Rural Fire Service NSW Bushfire Prone Lands dataset, which is publicly available (State Government of NSW and NSW Rural Fire Service, 2023).
Data collection	The data analyzed in this article includes behavioral information (clicks, webpage visits) that were collected automatically and were provided by the research partner (a large bank). The experiment was blinded as participants were unaware that multiple message versions existed and thus unaware of their own treatment assignment. Randomization, intervention delivery, and outcome logging were automated by the partner platform and therefore blind. Data analysis was not performed blind to the conditions of the experiments.
Timing	Data was collected between December 14, 2023 and January 9, 2024
Data exclusions	There were no post-data collection exclusions.
Non-participation	The bank had applied various exclusion criteria prior to sample selection (e.g. excluding customers who are minors, deceased, had previously opted out of marketing communications, participating in other bank campaigns, in hardship / arrears, guarantors). These customers were not part of the experiment, as pre-registered.
Randomization	Participants were randomly assigned to one of two conditions, receiving either generic communication (control, n = 6,524) or communication that included a proximal cue (treatment, n = 6,461). To check the validity of the random assignment, we compared the distribution of demographic and financial characteristics between conditions and found that the groups were indeed balanced (see Table 1 for a summary of these characteristics).

Reporting for specific materials, systems and methods

We require information from authors about some types of materials, experimental systems and methods used in many studies. Here, indicate whether each material, system or method listed is relevant to your study. If you are not sure if a list item applies to your research, read the appropriate section before selecting a response.

Materials & experimental systems

n/a	Involved in the study
☒	☐ Antibodies
☒	☐ Eukaryotic cell lines
☒	☐ Palaeontology and archaeology
☒	☐ Animals and other organisms
☒	☐ Clinical data
☒	☐ Dual use research of concern
☒	☐ Plants

Methods

n/a	Involved in the study
☒	☐ ChIP-seq
☒	☐ Flow cytometry
☒	☐ MRI-based neuroimaging

Plants

Seed stocks	NA
Novel plant genotypes	NA
Authentication	NA
